# A pan-genotypic indirect competitive ELISA for serological detection of pigeon circovirus antibodies

**DOI:** 10.3389/fmicb.2025.1612715

**Published:** 2025-07-30

**Authors:** Weifan Wang, Sa Xiao, Man Zhang, Jinming Liu, Jianxia Tian, Chuanyu Chang, Yuzhen Li, Yajie Zhang, Fuliang Zhang, Guiming Li, Xiaoyuan Yuan, Wenbin Wang

**Affiliations:** ^1^Poultry Institute, Shandong Academy of Agricultural Sciences, Jinan, China; ^2^College of Veterinary Medicine, Northwest A&F University, Yangling, China; ^3^Yangling Vocation and Technical College, Yangling, China; ^4^Shandong Provincial Key Laboratory of Livestock and Poultry Breeding (PKL2024B15), Jinan, Shandong, China

**Keywords:** indirect competitive ELISA, pigeon circovirus, Cap, virus-like particles, monoclonal antibody

## Abstract

Pigeon circovirus (PiCV) infection, which causes young pigeon disease syndrome (YPDS) and immunosuppression, significantly impacts both the meat and racing pigeon industries. Currently, no inactivated vaccine exists for PiCV prevention, primarily due to the challenges associated with isolating the PiCV virion, except for some gene subunit vaccines express the Cap protein of PiCV. The development of detection techniques is crucial for the diagnosis of PiCV. This study aimed to develop and validate a specific, sensitive indirect competitive enzyme-linked immunosorbent assay (icELISA) for detecting PiCV antibodies in pigeons. We identified the *cap* gene from a group C PiCV strain (PiCV/Shaanxi/China/10/2021, SX10) isolated from racing pigeons. The Cap of SX10, an immunogen, can self-assemble into virus-like particles (VLPs). A mouse monoclonal antibody (mAb) against Cap, 1G6-4C4, was selected to establish an icELISA. This mAb could identify the PiCV Cap of the strains in groups A to E. The pan-genotypic reactivity of mAb 1G6-4C4 might target a conserved conformational epitope, overcoming limitations of PCR and prior serological assays. The icELISA method exhibited no cross-reactivity with antibodies against other common pigeon pathogens, such as pigeon paramyxovirus type 1 (PPMV-1), avian influenza (H9N2), avian adenovirus type 4 (FAdV-4) or rotavirus (RV). Compared with indirect ELISA (iELISA), icELISA demonstrated comparable performance, as testing of 29 clinical serum samples revealed antibody-positive rates of 51.72% (icELISA) and 44.82% (iELISA), with a 93.10% concordance rate. To a certain extent, icELISA has demonstrated good specificity and sensitivity for detecting PiCV-specific antibodies in pigeons. The developed icELISA provides a robust, specific, and sensitive tool for the serological detection of PiCV infection. Complementary to PCR test, icELISA enhances the comprehensive detection of PICV in epidemiological studies by offering a more practical and sensitive alternative for field applications. Its utility for large-scale epidemiological surveillance in PiCV-endemic regions is validated, highlighting its potential to inform targeted biosecurity and control interventions.

## 1 Introduction

Pigeon circovirus (PiCV) is one of the factors that leads to young pigeon disease syndrome (YPDS), which causes immune organ atrophy and lymphocyte apoptosis (Raue et al., 2005; Todd, 2004). Additionally, PiCV infection may increase the pigeon’s susceptibility to other viruses or bacteria (Stenzel et al., 2012, 2014). At present, PiCV is prevalent in all regions of the world and is a common pathogen in pigeons (Freick et al., 2008; Zhang et al., 2015). Pigeons infected with PiCV exhibit nonspecific clinical symptoms, including anorexia, lethargy, depression, diarrhea, vomiting, crop effusion, rapid weight loss, ruffled feathers, and polyuria (Freick et al., 2008; Huang et al., 2017). The prevalence of PiCV infection can reach 70% depending on age and overall health status (Khalifeh et al., 2021). PiCV infection has caused huge economic losses worldwide and seriously endangered the development of the racing pigeon industry and meat pigeon breeding.

PiCV belongs to the genus *Circovirus* of the family *Circoviridae* (Mankertz et al., 2000). It is a non-enveloped icosahedral virus with a diameter of 15–18 nm; its genome is a single-stranded circular DNA (ssDNA) approximately 2.0 kb in size. The PiCV genome contains five open reading frames (ORFs): V1, C1, C2, the 3′ intergenic region, and the 5′ intergenic region (Gai et al., 2020). ORF V1 is located on the sense strand with ATG as the start codon, possesses 945–954 nucleotides and encodes replication-associated protein (Rep) with 314–317 amino acids (aa). ORF C1, is encoded in the antisense orientation with ATG/ATA as the start codon, possesses 813 to 834 nucleotides and encodes the capsid protein (Cap) with 270–277 aa, the only structural protein of the virus. The Cap protein is responsible for the assembly of the viral capsid and serves as the antigen that induces host antibody production during PiCV infection (Lai et al., 2014). Though Rep drives replication, Cap contains neutralizing antibody epitopes, inducing higher antibody titers than Rep in convalescent pigeon and during infection. Its high immunogenicity and structural conservation make it ideal for serodiagnosis and vaccine development (Stenzel et al., 2018; Gai et al., 2020). However, no commercial vaccines are currently available for PiCV (Loiko et al., 2018). PiCV is characterized by genetic diversity due to its high mutation rate during the replication cycle, and five major clades designated A to E, were identified based on nucleotide sequence analysis of the *cap* gene (Duffy and Holmes, 2008; Sarker et al., 2014). Clade A primarily consists of European isolates, with a small number of isolates from America and Asia. Clades B, C, and D include European and Asian isolates, and clade E includes isolates from the United States, Senegal, and Japan (Cságola et al., 2012; Yamamoto et al., 2015). Recent studies have further divided PiCV strains into additional clades based on whole-genome analysis (Li et al., 2024; Stenzel et al., 2024).

Asymptomatic infection with PiCV is quite common in meat and racing pigeons, with asymptomatic infection rates of 44% in Poland and California (Stenzel et al., 2014) and 63% in China (Zhang et al., 2015). Additionally, PiCV has not yet been successfully isolated *in vitro* due to the lack of a viable culture regimen for PiCV *in vitro*. Accurate diagnosis of PiCV is particularly important for detecting infected pigeons in a timely manner to control infection and viral spread. The detection methods used for PiCV are mainly molecular detection methods, including dot blot hybridization, conventional PCR, real-time PCR and loop-mediated isothermal amplification (Duchatel et al., 2006, 2009; Nath et al., 2022; Todd et al., 2002, 2006; Tsai et al., 2014). However, molecular detection methods can only detect the pathogen during the infection period. A full assessment of the PiCV infection status in pigeons requires serological methods to complement it. To date, only Stenzel et al. have developed and reported an indirect ELISA-based serological assay, demonstrating that ELISA serves as a highly effective complementary tool to molecular methods for assessing the PiCV infection status in domestic pigeons (Stenzel et al., 2017). But antibodies against pigeon IgG are scarce, and their specificity remains to be verified, that is, cross-reactions may exist. The icELISA methods usually have the characteristics of high sensitivity, strong specificity and weak cross-reaction.

In this study, an SX10 strain of PiCV was isolated from a racing pigeon loft in 2021. The whole-genome sequence and evolutionary relationships of SX10 were determined. We subsequently screened and prepared a mouse monoclonal antibody (mAb, 1G6-4C4) with specificity for the PiCV Cap. This mAb could identify the Cap from group A to E strains of P PiCV and was used as a competitive antibody to establish an icELISA method with high sensitivity and specificity compared with iELISA. Twenty-nine clinical samples were tested via icELISA. This method can accurately and rapidly detect PiCV-specific antibodies in pigeons, providing a foundation for the development of commercial serological detection kits.

## 2 Materials and methods

### 2.1 Ethics statement

All the animal experiments were approved by the Animal Care and Use Committee of Northwest A&F University, China, and conducted in strict accordance with the guidelines established by the Chinese Committee for Animal Experiments (Approval numbers: 2021ZX03011020-022 and 2023ZX0105010-002). Six-week-old specific pathogen-free (SPF) female BALB/c mice were purchased from Chong Qing Teng Xin Co., Ltd. (China). The mice were bled under anesthesia, and anesthesia was maintained with ketamine and xylazine. Every effort was made to minimize suffering. All the animals were euthanized with carbon dioxide.

### 2.2 Cells

The human embryonic kidney cell line HEK293T (ATCC CRL-11268), golden hamster kidney fibroblast line BHK-21 (ATCC CCL-10), and mouse myeloma (SP2/0) (ATCC CRL-1581) cells were cultured in Dulbecco’s modified Eagle’s medium (DMEM) supplemented with 10% fetal bovine serum (FBS) and 1% penicillin–streptomycin (P/S). All the cells were maintained at 37°C in a 5% CO_2_ atmosphere.

### 2.3 PiCV identification

PiCV infection was suspected in a racing pigeon loft in Shaanxi Province, China, in 2021. Four-week-old racing pigeons presented with depression, anorexia, diarrhea, and eventually death. The bursa of Fabricius of dead pigeons was collected and homogenized. Then, 400 μL of the supernatant was mixed thoroughly with 400 μL of chloroform and centrifuged at 12,000 rpm for 5 min. A total of 400 μL of the supernatant was collected. Then, 1 mL of anhydrous ethanol and 150 μL of sodium acetate were added, the mixture was mixed well. The mixture was then incubated for 30 min at −20°C. The DNA was dissolved in 20 μL of ddH_2_O after centrifugation at 12,000 rpm for 5 min, followed by washing with 75% ethanol. The RNA was extracted using RNAiso Reagen (Takara, Japan) according to the manufacturer’s instructions, and cDNA was synthesized from viral RNA using oligo(dT) primer with StarScript II First-strand cDNA Synthesis Mix (GenStar, China). The genes of interest, such as the F gene of Newcastle disease virus (NDV) (Standardization Administration of China, 2020), the polymerase gene of pigeon herpesvirus 1 (PiHV-1) (Raue et al., 2005), the fragment a of PiCV (Yu et al., 2007), and the VP6 gene of avian rotavirus A (AvRVA) (Harzer et al., 2021) were amplified from the DNA or RNA via PCR utilizing Phanta Max Super-Fidelity DNA Polymerase (Vazyme, China). The primers used to amplify fragments a and b (Table 1) were previously described by Yu et al. (2007, 2009). The primers used to amplify fragment c were designed according to the sequencing results of fragments a and b (Table 1). The following premixes were prepared according to the Phanta Max Super-Fidelity DNA Polymerase instructions: 25 μL of 2 × Phanta Max Buffer, 1 μL of dNTP mixure (10 mM each), 2 μL of forward and reverse primers (10 μM) and 1 μL of Phanta Max Super-Fidelity DNA Polymerase were mixed with 200 ng of template DNA in a total volume of 50 μL. The program consisted of the following step: 95°C for 3 min, followed by 35 cycles of denaturation at 95°C for 15 s, annealing at 58°C for 15 s, extension at 72°C for 30 s, and a final extension at 72°C for 5 min. The PCR products were visualized via 1% agarose gel electrophoresis and sequenced by Tsingke Biotech Co., Ltd. (Xi’an, China). The entire PiCV genome was assembled using SeqMan software and annotated with SnapGene 4.1.8. The complete genome of the isolate was submitted to GenBank (ID: OR573846.1), designated PiCV/Shaanxi/China/10/2021, and abbreviated SX10.

**Table 1:** Information about the primers used in this study.

Name	Target gene	Sequence (5′-3′)	Product size (bp)	References
NDV F	F	ATGGGCYCCAGAYCTTCTAC	535	Standardization Administration of China (2020)
NDV R		CTGCCACTGCTAGTTGTGATAATCC		
PiHV-1 F	Polymerase	GGGACGCTCTGATTAAGGAAT	242	Raue et al. (2005)
PiHV-1 R		CTTGGTGATCAGCAGCAGCTTG		
AvRVA F	VP6	CARCCWGCKCAYGATAATGTNTGTGG	596	Harzer et al. (2021)
AvRVA R		GTCCARTTCATWCCHGCWGGAAATACTGG		
PiCV a F	nt 140–159 (+)	GCTGCAGCTAAGCGATGGTG	630	Yu et al. (2007)
PiCV a R	nt 750–769 (−)	ACGCAGCAGCTCACAGAAGG		
PiCV b F	nt 1,648–1,667 (+)	GGTGTGCCCGAATCCTTTCC	774	Yu et al. (2009)
PiCV b R	nt 364–385 (−)	CTTCTCATTGTCCTCGTCACTG		
PiCV c F	nt 784–805 (+)	CCCGCATAAGGTGCCCGTGAAA	919	–
PiCV c R	nt 1,680–1,702 (−)	GGAAATGAGGCCAATTGGGGCCG		
pET30-Cap F	Cap	GGGGATCCATGAGAAGGCGGAGATTCTACCGCCGT	836	–
pET30-Cap R		GGGCGGCCGCTTCAGAATCAACAGCTGAGTCTGG		
FLAG-Cap F	Cap	CGCGGATCCATGAGAAGGCGGAGATTCTACCGCCGT	834	–
FLAG-Cap R		CCGGAATTCTTATTCAGAATCAACAGCTGAGTCTGG		

The underline represents the enzyme cleavage site. “–” indicates that the primers were designed in this study.

### 2.4 Phylogenetic analyses

A total of 856 complete nucleotide sequences of PiCV were collected from GenBank, and the detailed information is listed in Supplementary Table S1. For phylogenetic trees, the complete nucleotide sequences of PiCV and the amino acids Cap and Rep were first aligned with MAFFT v7.525 (Katoh and Standley, 2013), and the trees were constructed via IQ-TREE v2.3.6 (Minh et al., 2020). The GTR + G + I and LG + G + I models were used to construct nucleoside and amino acid phylogenetic trees, respectively. The accession numbers of the PiCV sequences used for phylogenetic analysis are shown in the phylogenetic trees. Images were generated via the Interactive Tree of Life (iTOL) website.^1^ Furthermore, a similarity analysis of the amino acid sequences of the Cap protein was performed using the Clustal W method with SDT v1.3 software (Muhire et al., 2014).

### 2.5 Preparation of the PiCV Cap antigen

The PiCV ORF C1 gene was amplified with Phanta^®^ Max Super-Fidelity DNA Polymerase (Vazyme, China). PCR was then performed using the primers described in Table 1. The PCR products were digested with the appropriate restriction enzymes and ligated into the pET-30a vector using T4 DNA ligase (TaKaRa, Japan) according to the manufacturer’s instructions. The constructed plasmid was transformed into BL21 competent cells (TaKaRa, Japan). Positive bacterial clones were cultured in LB media (1% tryptone, 0.5% yeast extract, 1% sodium chloride and 10 μg/mL kanamycin) and induced with 0.5 mM isopropyl-β-D-thiogalactoside (IPTG) for 6 h at 30°C. The cell pellets were collected, resuspended in lysis buffer (20 mM NaH_2_PO_4_, 500 mM NaCl, pH 8.0), and then lysed via ultrasonication. In accordance with the manufacturer’s instructions, soluble proteins in the supernatant were purified using Ni-NTA beads (Smart-Lifesciences, China), and the protein concentration was subsequently determined using a BCA protein assay kit (Solarbio, China). The prepared proteins were subjected to sodium dodecyl sulfate-polyacrylamide gel electrophoresis (SDS-PAGE) and Western blot analysis, and the morphology and size of the virus-like particles (VLPs) were observed via transmission electron microscopy (TEM).

### 2.6 TEM

The morphology and size of the VLPs self-assembled from Cap proteins in the concentrated protein mixture were observed via transmission electron microscopy (TEM). TEM and ultrastructural analysis were performed by Wuhan Servicebio Technology Co., Ltd. In brief, 20 μL of concentrated protein mixture was dropped onto a copper grid with a carbon film for 3–5 min, after which filter paper was used to absorb the excess liquid. Two percent phosphotungstic acid was added to the copper grid to stain for 1–2 min, filter paper was used to absorb excess liquid, and the sample was allowed to dry at room temperature. The cuprum grids were observed via TEM, and images were taken.

### 2.7 Production and characterization of mAbs specific for the PiCV Cap

Five 6-week-old SPF BALB/c female mice were immunized with purified PiCV Cap protein as an immunogen to prepare mAbs. For the first immunization, the mice were inoculated with a fully emulsified mixture of 50 μg protein and an equal volume of Freund’s complete adjuvant (Solarbio, China) via subcutaneous injection. After 21 days, a second booster immunization was performed using an immunogen emulsified with Freund’s incomplete adjuvant (Solarbio, China). At 35 and 40 days after the first immunization, the third and fourth booster immunizations were administered, with each mouse receiving an intraperitoneal injection of 50 μg of protein. After 3 days, serum from the mice was collected as a positive control serum for subsequent mAb screening, and serum collected before immunization was used as a negative control serum. The mice were euthanized after four immunizations. The collected splenocytes were then fused with SP2/0 cells according to standard procedures (Galfrè and Milstein, 1981), and the resulting hybridoma cells were screened using HAT and HT media. Hybridoma cells that secreted PiCV Cap antibodies were screened using indirect ELISA, immunofluorescence assay (IFA) and Western blotting. Positive hybridoma cells were subcloned twice by limited dilution to generate stable and effective cell lines that secreted specific mAbs for ascites production. Fifteen-week-old BALB/c mice were intraperitoneally injected with 1 × 10^6^ immunoreactive monoclonal cells, and ascites were collected multiple times over 10–14 days. The collected ascites was centrifuged to remove impurities, and the supernatant was collected. The mAb specificity was detected by IFA and Western blotting, and the subclass of the mAb was determined with a mouse mAb isotyping kit (Proteintech, China).

### 2.8 Indirect ELISA for evaluating Cap antibodies

To detect the titer of PiCV Cap antibodies in the serum of immunized mice and the titer of ascites mAbs and to screen for positive hybridoma cells secreting specific antibodies, an indirect ELISA was established as follows. Briefly, 96-well plates were coated with 100 μL of PiCV Cap protein (1 ng/μL) in coating buffer (0.05 M bicarbonate/carbonate, pH 9.6). The plates were washed with 200 μL of TBST (TBS buffer containing 0.05% Tween-20), blocked with 100 μL of blocking buffer (TBS solution containing 10% skim milk) and incubated for 2 h at 37°C. After washing, incubate with 100 μL of hybridoma cell supernatant or serum diluted with TBS buffer at 37°C for 1 h. The plates were washed and incubated with 100 μL of HRP-conjugated goat anti-mouse IgG (1:10000 dilution) at 37°C for 1 h. After washing, 100 μL of TMB substrate solution (Solarbio, China) was added for color development. The reaction was terminated with 50 μL of 2 M H_2_SO_4_ solution, and the absorbance at a wavelength of 450 nm was measured via a Spark multimode microplate reader (Tecan, United States).

### 2.9 IFA assay

The PiCV *cap* gene was subsequently cloned and inserted into the eukaryotic expression vector pcDNA3-2 × Flag (Table 1). The *cap* genes from the group A strain (GenBank No. PQ472729.1), group B strain (GenBank No. OQ715331.1), group D strain (GenBank No. OR843262.1) and group E strain (Genbank No. MW181971.1) were synthesized into pcDNA3-2 × Flag. A eukaryotic expression plasmid with a Flag tag was transfected into BHK-21 cells. After transfection for 36 h, the cells were fixed with 4% paraformaldehyde for 25 min and permeabilized with 0.1% Triton X-100 in PBS for 10 min at room temperature. After being blocked with PBS containing 1% bovine serum albumin (BSA) at 37°C for 2 h, the cells were incubated with the DDDDK-Tag mAb (ABclonal, China), hybridoma cell supernatant or the PiCV Cap protein mAb diluted in PBS at 37°C for 2 h. After being washed three times with PBS, the cells were incubated with goat anti-mouse IgG (Alexa Fluor® 594, ab150116, Abcam) at 37°C for 30 min. The cells were observed and imaged with an Olympus IX73 fluorescence microscope (Japan).

### 2.10 SDS-PAGE and Western blotting

The above eukaryotic expression plasmids were transfected into HEK293T cells, which were then lysed in ice-cold RIPA buffer containing the protease inhibitor phenylmethylsulfonyl fluoride (PMSF) (Solarbio, China) and boiled for 10 min for Western blot analysis. The purified PiCV Cap proteins were separated by SDS-PAGE and stained with Coomassie Brilliant Blue or transferred to polyvinylidene fluoride (PVDF) membranes (Millipore, United States). After the samples were blocked with TBS containing 10% skim milk, immunoblotting was performed with the following primary antibodies: mouse anti-His-Tag mAb (ABclonal, China), DDDDK-Tag mAb, hybridoma cell supernatant, or mouse PiCV Cap protein mAbs. HRP-conjugated goat anti-mouse IgG (H + L) (ABclonal, China) was used as the secondary antibody. An enhanced chemiluminescence (ECL) peroxidase substrate (Bio-Rad, United States) was used to detect proteins with the Tanon 5200 Chemiluminescent Imaging System (Tanon, China).

### 2.11 Establishment and optimization of indirect ELISA based on Cap protein

An indirect ELISA was established for detecting anti-PiCV antibodies. We incubated and maintained pigeons without PiCV infection, as indicated by multiple rounds of PCR in a clean environment. We collected the blood from pigeons in three rounds, with an interval of 2 weeks each time. Genomic DNA was extracted using TIANamp Genomic DNA Kit (TIANGEN, China). As mentioned above, PCR was performed using the DNA of blood samples as templates and PiCV a F&R in Table 1 as primers. If all three PCR tests are negative, it is determined that the pigeon is not infected with PiCV. These serum of the pigeons were used as PiCV antibody-negative serum. The amount of antigen, used to coat the plates and the dilution of the serum samples were determined via checkerboard titration. The antigen coating concentration and serum dilution were determined when the ratio of the OD_450nm_ value of the positive serum to that of the negative serum (P/N) was maximized, and these conditions were used as optimal. After the above parameters were determined, the indirect ELISA procedure was as follows. 100 μL of purified protein was incubated in 96-well plates in coating buffer at 4°C overnight. After the plates were washed with 200 μL of TBST three times, they were blocked with 100 μL of blocking buffer at 37°C for 2 h. After washing three times, 100 μL of the serum samples were added to the plates and incubated at 37°C for 60 min. After washing three times, 100 μL of goat anti-bird IgY H&L (HRP, ab112773, Abcam) was added to each well and incubated at 37°C for 45 min. After washing three times, 100 μL of the TMB substrate solution was added to induce the colorimetric reaction. The reaction was terminated with 50 μL of 2 M H_2_SO_4_ solution and the absorbance at a wavelength of 450 nm was measured. The concentration of the blocking solution and the incubation time of the serum were subsequently optimized. The dilution ratio and incubation time of the second antibody were further optimized, and finally, the TMB reaction time was optimized. The highest P/N value was used as the basis for selecting optimal conditions. Each sample was repeated three times on the same or different batches of microplates.

### 2.12 Validation of indirect ELISA based on Cap protein

To determine the cut-off value for the indirect ELISA, 32 anti-PiCV antibody-negative pigeon serum samples were tested via the indirect ELISA developed here, and the cut-off value for the positive results was calculated on the basis of the average OD_450nm_ value of the 32 negative serum samples plus 3 standard deviations (SDs) to ensure 99% confidence in a negative serological result obtained within this range. To confirm the specificity of the indirect ELISA, pigeon serum samples positive for anti-pigeon paramyxovirus type 1 (PPMV-1), anti-avian influenza (H9N2), avian adenovirus type 4 (FAdV-4) and rotavirus (RV) antibodies that our laboratory preserved were tested via indirect ELISA. The sensitivity of the indirect ELISA was assessed by detecting pigeon serum that was positive for anti-PiCV antibodies. To evaluate the reproducibility of the indirect ELISA, the coefficient of variation (CV) for inter-assay variation (between plates) and intraassay variation (within a plate) was determined.

### 2.13 Establishment and optimization of icELISA based on PiCV Cap protein mAb

The coating antigen concentration and dilution of the PiCV Cap protein mAb were determined via checkerboard titration. We incubated and maintained pigeons without PiCV infection, as indicated by multiple rounds of PCR in a clean environment. The serum of the pigeons was used as PiCV antibody-negative serum. These clean pigeons were inoculated subcutaneously with 80 μg of purified Cap protein, followed by a booster injection 7 days later. Serum was collected 14 days after the initial injection as PiCV antibody-positive serum. As the OD_450nm_ value of the negative serum samples was slightly greater than 1.0, the optimal amount of coated antigen and the optimal mAb dilution were selected based on the ratio of the OD_450nm_ value of the negative serum to that of the positive serum. After the above parameters were determined, the indirect competitive ELISA procedure was as follows. 100 μL of purified protein was incubated in 96-well plates in coating buffer at 4°C overnight. After the plates were washed three times with 200 μL of TBST, they were blocked with 100 μL of blocking buffer at 37°C for 2 h. After washing three times, 100 μL of serum samples were added to the plates and incubated at 37°C for 80 min. After washing three times, 100 μL of diluted mAb was added to the plates and incubated at 37°C for 60 min. After washing three times, 100 μL of HRP-conjugated goat anti-mouse IgG was added to each well, and the mixture was incubated at 37°C for 45 min. After washing three times, 100 μL of TMB substrate solution was added to induce the colorimetric reaction. The reaction was terminated with 50 μL of 2 M H_2_SO_4_ solution and the absorbance at a wavelength of 450 nm was measured.

Based on this, the optimal dilution of the serum samples was determined. The concentration of blocking solution, serum incubation time and mAb incubation time were subsequently optimized. The dilution ratio and incubation time of the second antibody were further optimized, and finally the TMB reaction time was optimized. The percentage of inhibition (PI) was calculated via the following formula: PI (%) = [1-(OD_450nm_ value of test serum samples/OD_450nm_ value of negative serum samples)] × 100%. The highest PI value was used as the basis for selecting the condition. Each sample was repeated three times on the same or different batches of microplates.

### 2.14 Validation of icELISA

To determine the cut-off value for the icELISA, 32 anti-PiCV antibody-negative pigeon serum samples were tested via the icELISA developed here, and the cut-off value for the positive results was calculated on the basis of the average PI of the 32 negative serum samples plus 3 standard deviations (SDs) to ensure 99% confidence in obtaining a negative serological result within this range. To confirm the specificity of the icELISA, pigeon serum samples positive for anti-PPMV-1, anti-avian influenza (H9N2), FAdV-4 and RVA antibodies that our laboratory preserved were tested via icELISA. The sensitivity of the icELISA was assessed by detecting pigeon serum that was positive for anti-PiCV antibodies. To evaluate the reproducibility of the icELISA, the coefficient of variation (CV) for inter-assay variation (between plates) and intra-assay variation (within a plate) was determined.

### 2.15 Statistical analysis

All experimental data were analyzed via two-tailed Student’s *t*-tests via GraphPad Prism 8.0 software (GraphPad Inc., San Diego, CA). Data from three independent experiments are presented as the means ± standard deviations (SDs) from triplicate samples (*n* ≥ 3).

## 3 Results

### 3.1 Identification of PiCV in a racing pigeon loft

**Figure 1:**
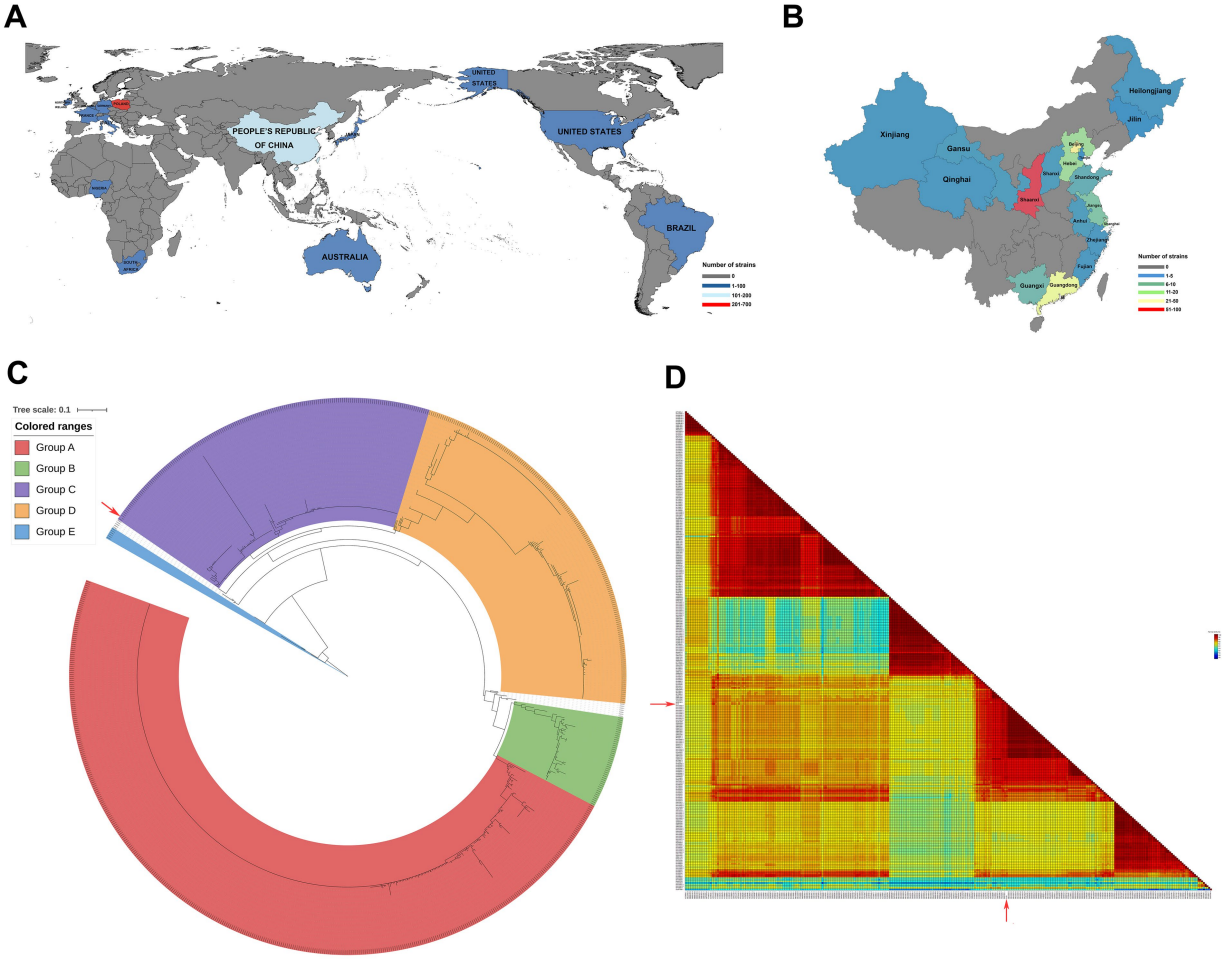
Geographical distribution of PiCV and phylogenetic and homology analyses of PiCV. All the PiCV strains from GenBank were analyzed for geographical distribution worldwide **(A)** and in China **(B)**. **(C)** Phylogenetic trees based on the amino acids of Cap. The trees were aligned with MAFFT v7.525 and constructed via IQ-TREE v2.3.6. **(D)** Pairwise identity matrices calculated with the ClustalW method comparing the percentage of amino acid identity among SX10 and the 296 PiCV Cap. The isolates used in this study are indicated by a red solid arrow. SX10, PiCV/Shaanxi/China/10/2021.

Currently, PiCV is popular in thirteen countries worldwide, particularly in Poland and China (Figure 1A). In China, it is particularly precvalent in 18 provinces, with Shaanxi Province being a notable example (Figure 1B). Here, DNA and RNA extracted from dead racing pigeons in Shaanxi Province were used as templates for PCR, and the resulting band was sequenced and used for sequence alignment to confirm that it originated from the PiCV genome (Supplementary Figure S1). The NDV F gene band was amplified successfully via the NDV universal primer (Table 1). The target band of NDV was the live poultry vaccine LaSota because of pigeon immunization. The whole PiCV genome sequence of the strain was assembled via SeqMan software and designated PiCV/Shaanxi/China/10/2021 (SX10) under GenBank accession number OR573846.1. The full-length genome size of SX10 was 2,036 bp.

To investigate the evolutionary relationships between SX10 and all the other PiCV strains, phylogenetic trees based on the complete nucleotide sequences (Supplementary Figure S2A) and the animo acids Cap (Figure 1C) and Rep (Supplementary Figure S2B) were constructed. PiCV can be divided into groups A–E, and the SX10 strain belongs to Group C (Figure 1C). The amino acid sequences of Cap from the 296 PiCV strains in GenBank were analyzed via the SDT v1.3 software. The amino acid identity of Cap between the SX10 and 296 PiCV strains ranged from 71.69 to 98.20%, with an average value of 86.29% (Figure 1D).

### 3.2 Preparation of the Cap antigen

**Figure 2:**
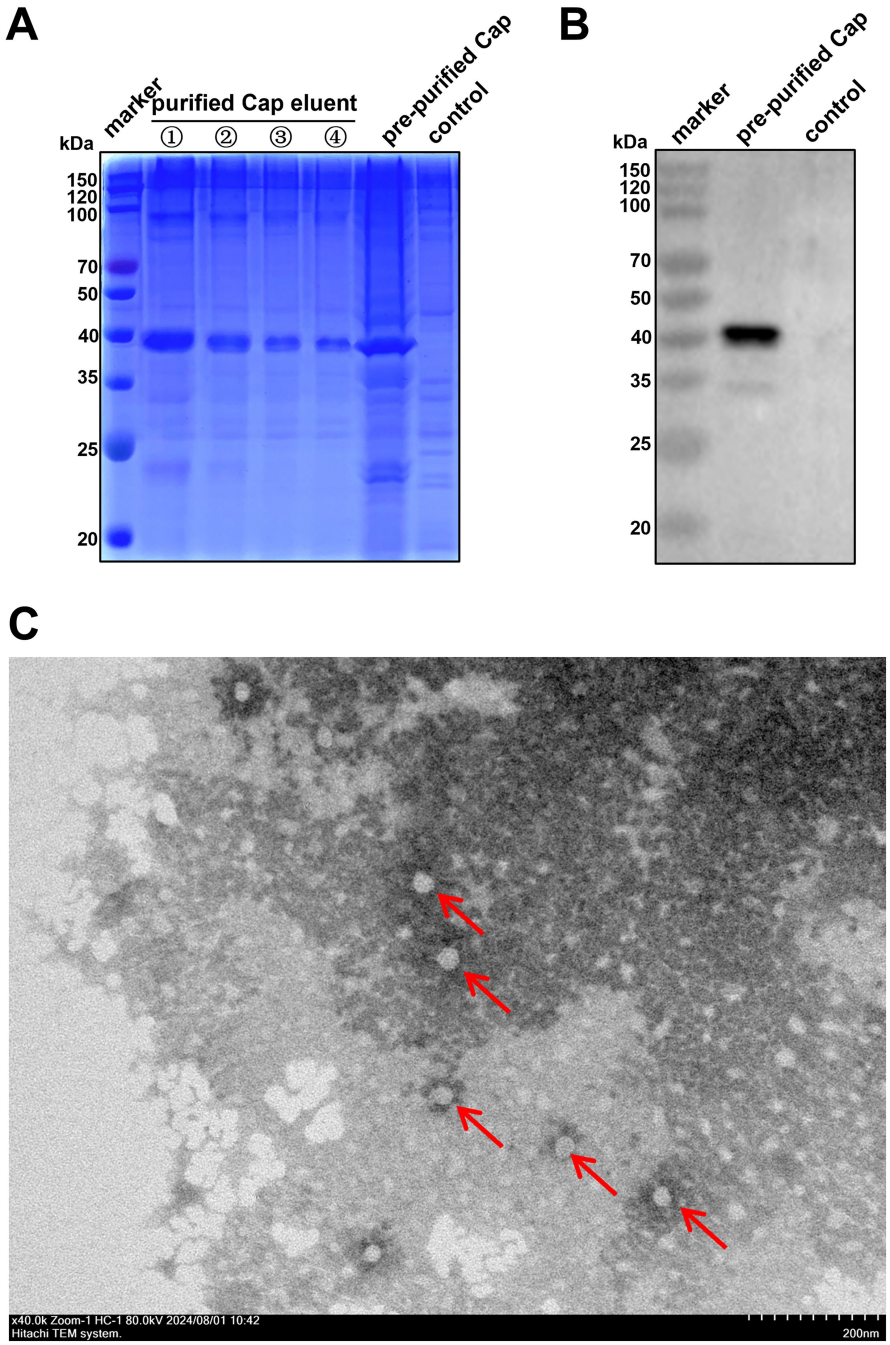
Purification of the PiCV Cap. **(A)** SDS-PAGE assay of the expression and purification of the PiCV Cap. **(B)** The purified PiCV Cap protein was detected using a Western blotting with a mouse anti-His-tag mAb. **(C)** VLPs self-assembled from Cap, as observed via TEM (red arrows).

Recombinant SX10 Cap with a molecular weight of approximately 40 kDa was successfully expressed via a prokaryotic expression system (Figure 2A). The purified recombinant Cap was obtained using a nickel column and detected with a mouse anti-His-Tag mAb (Figure 2B). TEM revealed that this purified Cap protein self-assembled into VLPs with diameters of approximately 40 nm (Figure 2C), indicating that the Cap expressed in prokaryotic cells had self-assembly activity and good immunogenicity.

### 3.3 Identification of mAbs specific for Cap

**Figure 3:**
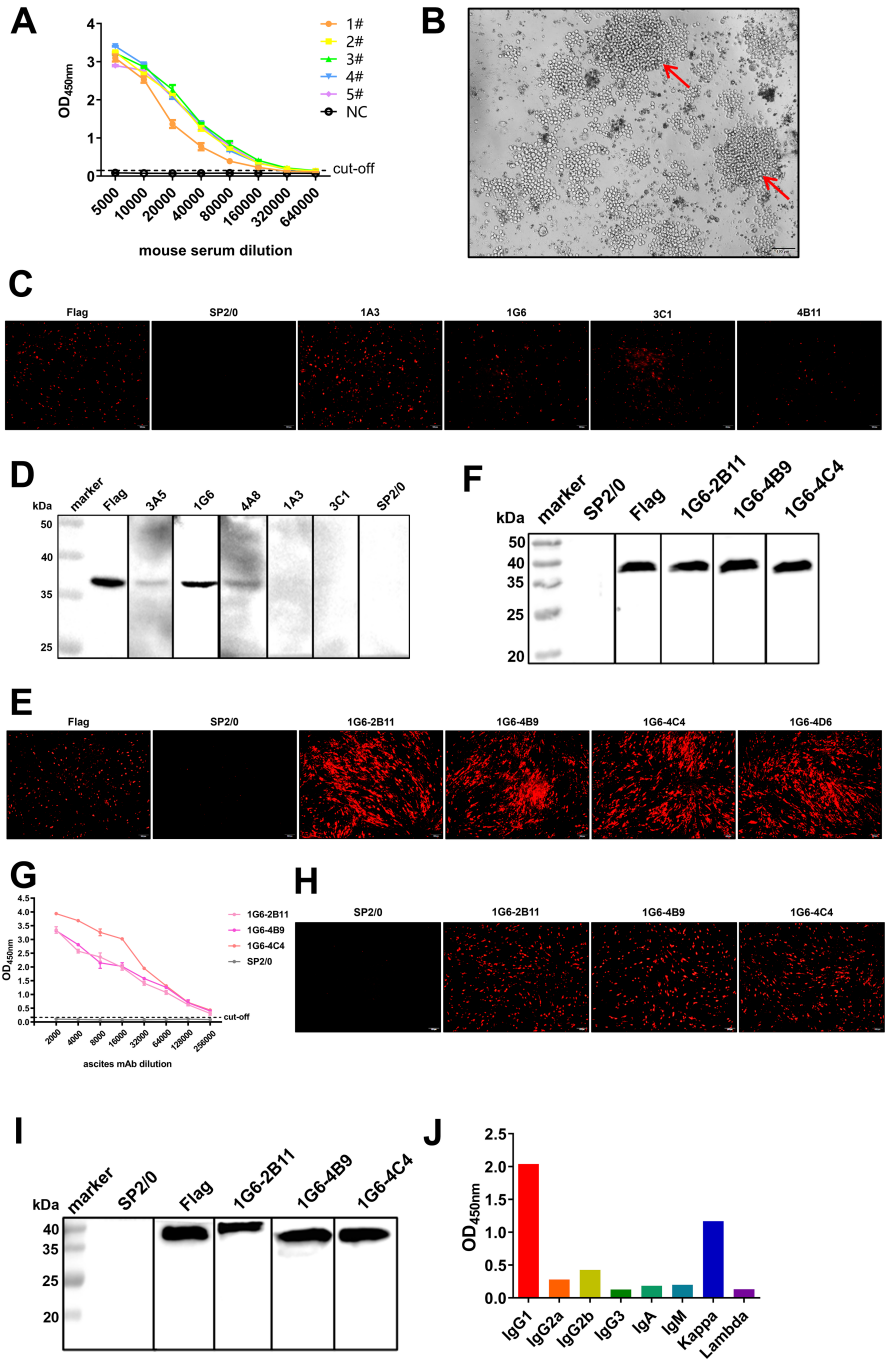
Production, screening and characterization of Cap mAbs. **(A)** The serum antibody titers of five BALB/c mice immunized with the PiCV Cap protein were detected via iELISA. NC, negative control. **(B)** The splenocytes of immunized mice were fused with SP2/0 cells to form grape-string cells (red arrows). IFA assay and Western blotting of hybridoma cells **(C,D)** and monoclonal cells **(E,F)**. A mouse anti-DDDDK-Tag mAb was used as a positive control, and the supernatant of SP2/0 cells was used as a negative control. **(G)** Ascites mAb titers were determined by indirect ELISA. All representative data from three independent experiments (mean ± SD) were analyzed with a two-tailed Student’s *t*-test. IFA assay **(H)** and Western blotting **(I)** for mAbs. **(J)** The subclass of the mAb 1G6-4C4 was determined with a mouse mAb isotyping kit.

The purified SX10 Cap was used as an immunogen to immunize mice, resulting in the production of high levels of antibodies suitable for hybridoma cell preparation. The titers of the polyclonal antibodies (pAbs) in 5 mice after immunization were determined using iELISA, which revealed a satisfactory immune response (Figure 3A). The hybridoma cells formed grape clusters on day 8 after cell fusion in the cell culture plate (Figure 3B). Preliminarily, the supernatants of hybridoma cells 1A3, 1G6, 3C1, and 4B11, which were used as primary antibodies for evaluating the eukaryotic expression of Cap in BHK-21 cells were positively screened via an IFA (Figure 3C). The antibodies secreted by 3A5, 1G6, and 4A8 specifically bound to Cap, as shown via Western blotting (Figure 3D). No fluorescence was observed in the negative control SP2/0. Notably, the hybridoma cell line 1G6 reacted with Cap, as shown by both IFA and Western blotting. After two rounds of subcloning, 1G6-2B11, 1G6-4B9, and 1G6-4C4 monoclonal cells were positive according to both IFA (Figure 3E) and Western blotting (Figure 3F). The ascites mAbs of these three types of monoclonal cells were produced successfully, and the titer of 1G6-4C4 was the highest, reaching 1 × 10^5^ (Figure 3G). Specific red fluorescence was observed after incubation with these three mAbs, whereas no fluorescence was observed in negative ascites (Figure 3H). However, after incubation with 1G6-2B11, the mass of Cap increased (Figure 3I). Above, we selected 1G6-4C4 for the follow-up study. The heavy chain subclass of 1G6-4C4 was IgG1, and the light chain subtype was kappa (Figure 3J), indicating that it was a suitable secretory mAb. We found that 1G6-4C4 could recognize strains from A-E of PiCV (Supplementary Figure S3). These results indicated that the mAb 1G6-4C4 was an ideal antibody for detecting PiCV.

### 3.4 Optimization and evaluation of icELISA specificity, sensitivity, and reproducibility

**Figure 4:**
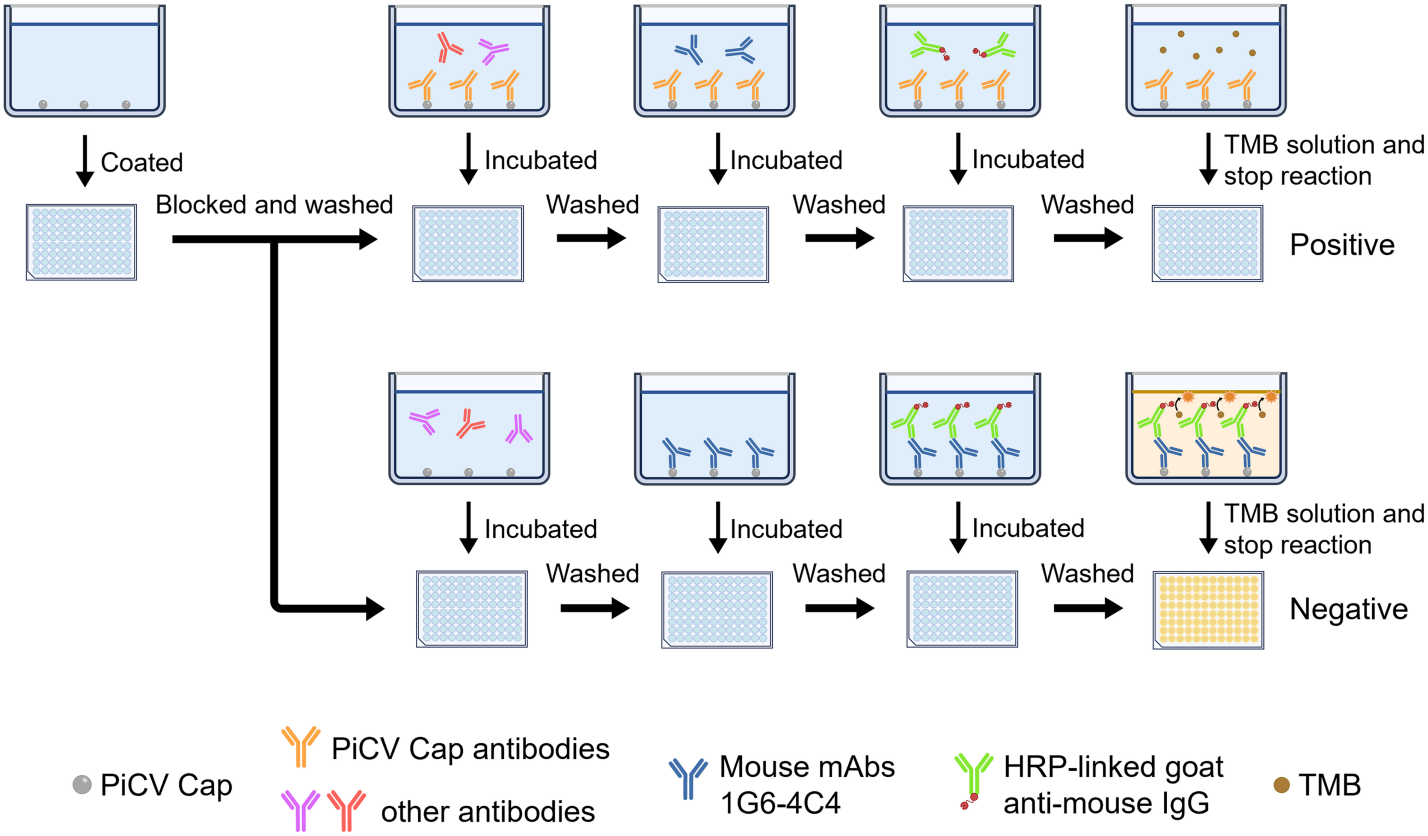
Flowchart of icELISA establishment.

**Figure 5:**
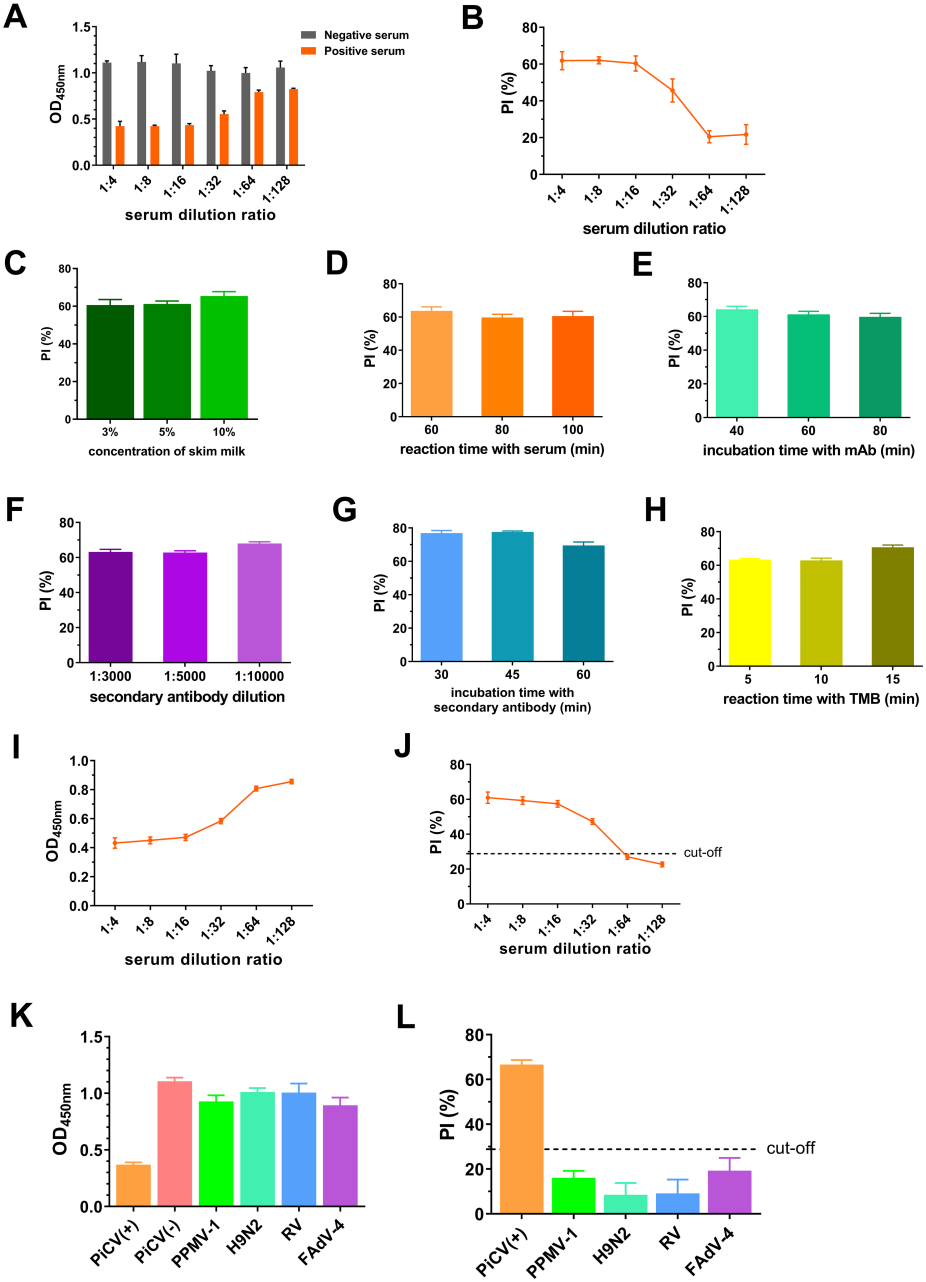
Establishment and optimization of icELISA. OD_450nm_ **(A)** and PI values **(B)** of serum at different dilutions. PI values with different concentration of skim milk in blocking buffer **(C)**, reaction time with serum **(D)**, incubation time with mAbs **(E)**, dilution of secondary antibodies **(F)**, incubation time with secondary antibodies **(G)** and reaction time with TMB **(H)**. OD_450nm_ **(I)** and PI **(J)** of PiCV positive serum samples at different dilutions. OD_450nm_ **(K)** and PI **(L)** of different viral serum-positive antibodies. Representative data from three independent experiments (mean ± SD) were analyzed with a two-tailed Student’s *t*-test.

According to the flowchart for icELISA establishment (Figure 4), the optimal antigen coating concentration and dilution of 1G6-4C4 were determined by checkerboard titration to be 50 ng/well and 1:8000, respectively (Table 2). Using a higher PI value as the basis for selection, the optimal serum sample dilution was determined to be 1:8 (Figures 5A,B). The optimal parameters of icELISA were as follows: the concentration of skim milk in the blocking buffer was 10% (Figure 5C), the reaction time with the serum sample was 60 min (Figure 5D), the incubation time with the mAb was 40 min (Figure 5E), the dilution of the secondary antibody was 1:10000 (Figure 5F), the incubation time with the secondary antibody was 45 min (Figure 5G), and the reaction time with TMB was 15 min (Figure 5H). The cut-off values for positive results were determined via 32 negative serum samples. The results revealed that the mean PI value and standard deviation (SD) of the 32 serum samples were 7.46 and 7.12%, respectively, and the icELISA cut-off value was 28.83% (mean +3 SD). Therefore, the pigeon serum samples with PIs ≥ 28.83% were positive, and those with PIs < 28.83% were negative. The PiCV antibody-positive serum remained positive when diluted to 1:32, indicating good icELISA sensitivity (Figures 5I,J). This icELISA was negative for PPMV-1 antibody-positive serum, H9N2 antibody-positive serum, FADV-4 antibody-positive serum, and RV antibody-positive serum, indicating that the established icELISA had good specificity (Figures 5K,L). These serum samples were tested using the same batch and different batches of plates, and the results revealed that the intra-batch and inter-batch coefficients of variation were less than 10% (Supplementary Tables S2, S3), indicating that the icELISA had good repeatability.

**Table 2:** Determination of the optimal amount of antigen coating and dilution of 1G6-4C4 for icELISA.

Antigen coating amount^a^		Monoclonal antibody dilution					
		1:2000	1:4000	1:8000	1:16000	1:32000	1:64000
25 ng	N	1.146 ± 0.132	0.889 ± 0.048	0.799 ± 0.095	0.720 ± 0.073	0.694 ± 0.053	0.527 ± 0.054
	P	0.317 ± 0.015	0.257 ± 0.003	0.215 ± 0.009	0.149 ± 0.006	0.105 ± 0.020	0.070 ± 0.001
	N/P	3.621	3.466	3.723	4.832	6.641	7.521
50 ng	N	1.576 ± 0.129	1.311 ± 0.038	1.032 ± 0.001	0.946 ± 0.035	0.823 ± 0.020	0.604 ± 0.096
	P	0.503 ± 0.053	0.420 ± 0.003	0.336 ± 0.002	0.257 ± 0.009	0.200 ± 0.001	0.145 ± 0.019
	N/P	3.133	3.121	3.070	3.688	4.113	4.176
100 ng	N	1.708 ± 0.205	1.499 ± 0.020	1.217 ± 0.054	1.036 ± 0.083	0.943 ± 0.013	0.739 ± 0.032
	P	0.875 ± 0.081	0.646 ± 0.019	0.539 ± 0.016	0.436 ± 0.014	0.289 ± 0.002	0.188 ± 0.001
	N/P	1.953	2.320	2.259	2.375	3.269	3.941
200 ng	N	2.302 ± 0.161	1.900 ± 0.118	1.609 ± 0.099	1.390 ± 0.066	1.004 ± 0.011	0.742 ± 0.053
	P	1.942 ± 0.008	1.565 ± 0.001	1.321 ± 0.006	0.980 ± 0.016	0.619 ± 0.008	0.364 ± 0.025
	N/P	1.185	1.214	1.218	1.418	1.622	2.041

a When the OD_450nm_ value of negative serum (N) samples is slightly greater than 1.0 and the ratio of the OD_450nm_ value of negative serum that of positive serum (N/P) is the largest, the optimal condition is considered.

### 3.5 Optimization and validation of iELISA

**Figure 6:**
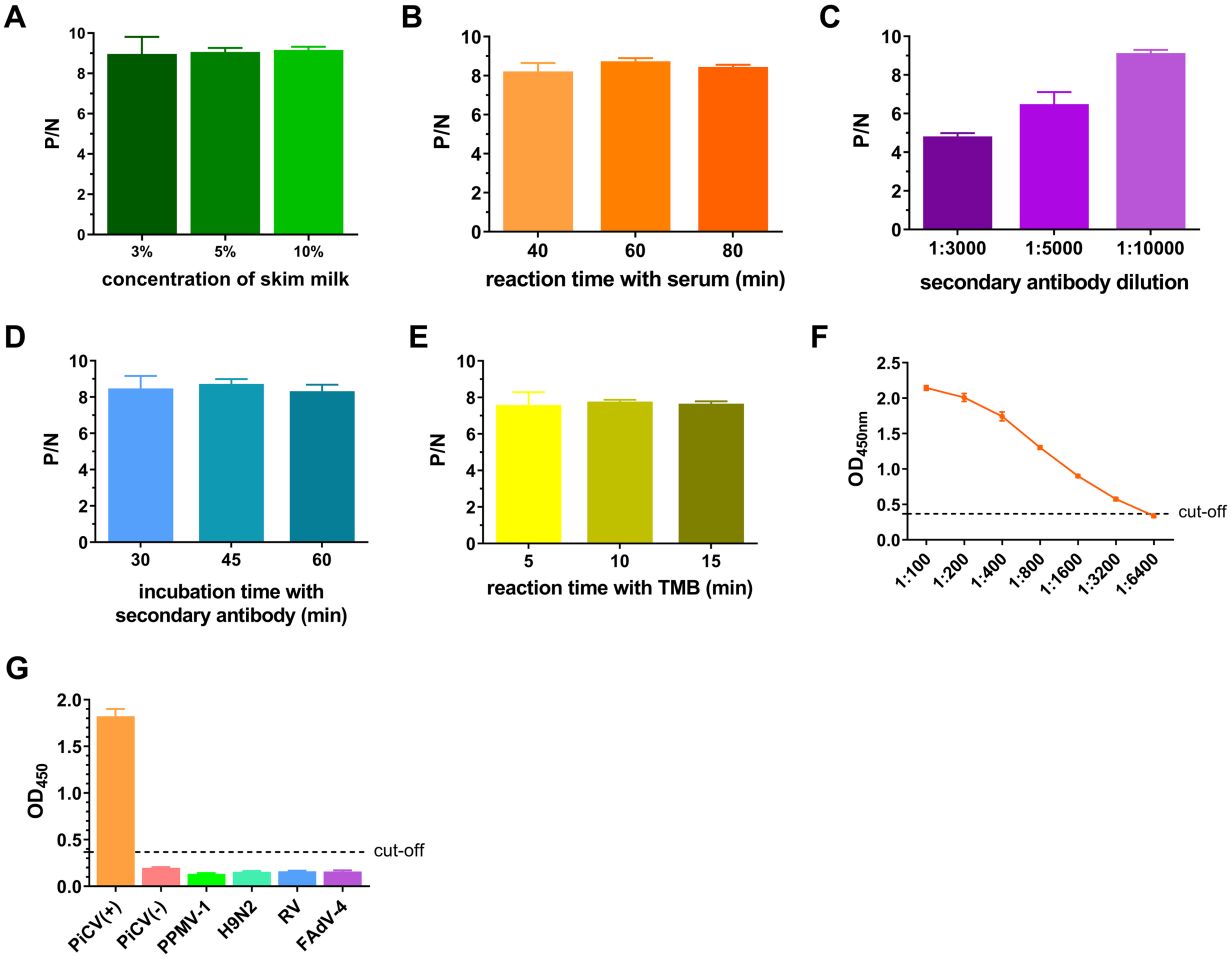
Establishment and optimization of iELISA. The ratio of the OD_450nm_ of the positive serum (P) to that of the negative serum (N) with different concentrations of skim milk used for blocking **(A)**, the reaction time with the serum **(B)**, the dilution of the secondary antibody **(C)**, the incubation time with the secondary antibody **(D)** and the TMB reaction time **(E)**. **(F)** OD_450nm_ values of serum samples at different dilutions. **(G)** OD_450nm_ values of different virus-positive serum samples. All representative data from three independent experiments (mean ± SD) were analyzed with a two-tailed Student’s *t*-test.

Given the lack of a commercially available PiCV ELISA kit, iELISA was established as the control. The optimal antigen coating concentration and the optimal dilution of serum samples for iELISA were 200 ng/well and 1:400, respectively (Table 3). The optimal conditions were as follows: the concentration of skim milk was 10% (Figure 6A), the reaction time of the serum sample was 60 min (Figure 6B), the dilution of the secondary antibody was 1:10000 (Figure 6C), the incubation time of the secondary antibody was 45 min (Figure 6D), and the TMB reaction time was 10 min (Figure 6E). The cut-off values for iELISA-positive results were determined via 32 pigeon-negative serum samples. The results revealed that the mean OD_450nm_ value and standard deviation (SD) of the 32 serum samples were 0.179 and 0.063, respectively, and the indirect ELISA cut-off value was 0.368 (mean +3 SD). Therefore, pigeon serum samples with OD_450nm_ ≥ 0.368 were positive, and those with OD_450nm_ < 0.368 were negative. The PiCV antibody-positive serum remained positive when diluted to 1:3200, indicating good iELISA sensitivity (Figure 6F). The PiCV antibody detection results for the different viral serum samples were negative, indicating that the established iELISA had good specificity (Figure 6G). The intra-batch and inter-batch coefficients of variation of these serum samples were less than 10% (Supplementary Tables S4, S5), indicating that iELISA had good repeatability.

**Table 3:** Determination of the optimal amount of antigen coating and the optimal dilution of serum for iELISA.

Antigen coating amount^a^		Serum dilution					
		1:100	1:200	1:400	1:800	1:1600	1:3200
	P	0.794 ± 0.090	0.673 ± 0.032	0.649 ± 0.129	0.514 ± 0.078	0.354 ± 0.047	0.182 ± 0.002
25 ng	N	0.232 ± 0.020	0.180 ± 0.004	0.098 ± 0.007	0.080 ± 0.005	0.065 ± 0.007	0.065 ± 0.003
	P/N	3.428	3.736	6.651	6.459	5.481	2.822
	P	0.952 ± 0.016	0.819 ± 0.019	0.719 ± 0.027	0.523 ± 0.002	0.397 ± 0.011	0.228 ± 0.008
50 ng	N	0.255 ± 0.010	0.214 ± 0.001	0.119 ± 0.003	0.101 ± 0.002	0.069 ± 0.002	0.058 ± 0.001
	P/N	3.739	3.827	6.063	5.173	5.796	3.957
	P	1.640 ± 0.047	1.502 ± 0.025	1.300 ± 0.013	0.960 ± 0.014	0.642 ± 0.012	0.373 ± 0.001
100 ng	N	0.282 ± 0.003	0.215 ± 0.007	0.134 ± 0.002	0.120 ± 0.005	0.108 ± 0.021	0.070 ± 0.008
	P/N	5.824	6.984	9.701	8.033	5.940	5.321
	P	2.123 ± 0.055	2.008 ± 0.053	1.743 ± 0.071	1.291 ± 0.008	0.897 ± 0.009	0.541 ± 0.011
200 ng	N	0.329 ± 0.011	0.246 ± 0.004	0.148 ± 0.005	0.120 ± 0.013	0.104 ± 0.011	0.097 ± 0.002
	P/N	6.461	8.177	11.774	10.758	8.667	5.601

a When the ratio of the OD_450nm_ value of positive serum to negative serum (P/N) is highest, the optimal condition is considered.

### 3.6 Evaluation of clinical pigeon samples with icELISA and iELISA

Twenty-nine clinical racing pigeon serum samples were collected and valuated via icELISA and iELISA. Fifteen and thirteen of the twenty-nine samples were positive according to icELISA and iELISA, respectively (Supplementary Tables S6, S7). Among the 15 positive samples detected with icELISA, two samples were negative with iELISA, and the results of the remaining samples were consistent (Table 4). The coincidence rate for clinical samples evaluated with icELISA and iELISA was 93.10% (27/29). This result indicated that icELISA was more accurate than iELISA to a certain extent.

**Table 4:** Comparison of the results of icELISA and iELISA.

	icELISA	iELISA
Positive No.	15	13
Negative No.	14	16
Positive rate	51.72%	44.82%
Coincidence rate	93.10%	

## 4 Discussion

Currently, strategies employed to prevent and control PiCV primarily focus on detection and removal. Most methods for detecting PiCV are laboratory detection methods, such as qPCR assays. Serological detection techniques are lacking. In this study, a group C strain of PiCV was identified in racing pigeons. A mAb, 1G6-4C4, was subsequently screened and could specifically recognize PiCV Cap from groups A to E. An icELISA was established using 1G6-4C4 to detect PiCV antibodies. Compared with iELISA, icELISA demonstrates promising specificity and accuracy. Notably, two samples that tested negative in iELISA yielded positive results in icELISA. This discrepancy may be attributed to the higher standard deviation observed in iELISA during the establishment of judgment criteria, which could lead to an increased number of samples being classified as suspicious in clinical testing. The goat anti-bird IgY was used to establish iELISA, which might have a poor specific binding with pigeon serum, thereby resulting in false negative results. The icELISA has better specificity and is more accurate. The icELISA is precise and convenient for detecting PiCV-specific antibodies in pigeons.

PiCV infection in pigeons is a common occurrence. According to previously reported statistics, asymptomatic PiCV infection was detected in 62.5% of pigeons in eastern China in 2009, with a total PiCV-positive rate of 75% (Zhang et al., 2011). The data for 2014 revealed that these values were 63.3 and 75.3%, respectively (Zhang et al., 2015), indicating that PiCV is widely distributed and that the infection rate remains high in meat pigeons. With the gradual rise of pigeon racing and the improvement of competition rules, pigeons from diverse breeding environments and varying health conditions are housed together in the same shelter to ensure fair competition results. This situation promotes the spread of various pathogens, making subclinical PiCV infection quite common, with an estimated prevalence of approximately 40% in pigeons (Stenzel et al., 2017, 2019; Khalifeh et al., 2021; Stenzel et al., 2024). Pigeons are also commonly used as experimental animal models in scientific research; however, latent infection with PiCV renders them unsuitable for use as animal models (Li et al., 2024).

Previously, VLPs generated via the self-assembly of Cap using baculovirus expression system were shown to be immunogenic and capable of inducing specific antibodies in mice (Gai et al., 2020). Here, Cap expressed via *E. coli* expression systems could also self-assemble into VLPs (Figure 2C). The lack of isolation and culture methods for PiCV *in vitro* limits the strategy of screening hybridoma cells by infecting host cells with viruses. In this study, eukaryotically expressed labeled Cap was used for antibody screening. In addition, the specific binding sites of the mAbs screened in this study for Cap and their conservation require further experimental investigation.

Serological detection is a simple and inexpensive method for rapid large-scale screening, and it is widely used in veterinary practice, particularly for diagnosing infectious diseases. The ELISA technique is most commonly used in veterinary serological testing because it has the following characteristics: simple operation, high throughput, a high degree of automation, and high sensitivity and specificity. The anti-mouse secondary antibodies used in icELISA are less expensive than the anti-bird secondary antibodies used in iELISA. Additionally, the specificity of anti-bird antibodies across avian species remains unclear. In general, icELISA is more sensitive and suitable for commercial development than iELISA is. Considering the limited blood collection of racing pigeons, icELISA in this research requires slightly more serum volume than iELISA does, which is the disadvantage of icELISA. Since the clinical serum samples used in this study primarily came from racing pigeons in Shaanxi Province, the applicability of this method may be limited. The serum samples of meat pigeons, carrier pigeons, and racing pigeons from more provinces and regions in China will be further assessed. Notably, the serological assay for asymptomatic carriers, which is the presence of a positive pathogen test result in the absence of clinical manifestations, is a key challenge in contemporary pathogen detection research. Serological assay provides indirect evidence of prior infection or immune status. However, not all pathogen infections result in antibody production in individuals, as exemplified by SARS-CoV-2. Consequently, serological testing might fail to detect certain asymptomatic carriers. Currently, no vaccine is available for PiCV. The implementation of real-time PCR combined with serological analysis provides an effective strategy for the detection and control of PiCV.

In conclusion, we screened and identified an mAb that is specific to the PiCV Cap. An icELISA method was established based on this PiCV mAb to detect PiCV antibodies in pigeons. The developed assay effectively detects PiCV antibodies. This method, which has high specificity for detecting PiCV-specific antibodies in pigeons, shows promise for epidemic surveillance and serum evaluation.

## 5 Conclusion

This study successfully established an icELISA for the serological surveillance of PiCV, a pathogen that critically impacts global pigeon industries through immunosuppression and YPDS. By leveraging the self-assembling Cap protein of the PiCV/Shaanxi/China/10/2021 (group C) into conformationally intact VLPs, we generated a pangenotypic mAb, 1G6-4C4, with broad reactivity against PiCV strains in groups A to E. The icELISA exhibited good specificity, showing no cross-reactivity with PPMV-1, H9N2, FAdV-4, and RV, and demonstrated superior sensitivity compared to the iELISA in field testing. The icELISA detected PiCV-specific antibodies (51.72%) in cases (*n* = 29), outperforming the iELISA (44.82%), with 93.10% concordance. These findings underscore its potential to guide targeted biosecurity interventions.

## Data Availability

The datasets presented in this study can be found in online repositories. The names of the repository/repositories and accession number(s) can be found below: https://www.ncbi.nlm.nih.gov/genbank/, OR573846.1.

## Footnotes

^1^ http://itol.embl.de/

## Supplementary material

Supplementary Figure S1 PCR amplification of target genes. **(A)** PCR amplification of viral genes. NDV, Newcastle disease virus; PiHV-1, pigeon herpesvirus 1; PiCV, pigeon circovirus; AvRVA, avian rotavirus A. “-” indicates that the primers were designed in this research. **(B)** PCR amplification of PiCV gene fragments.

Supplementary Figure S2 Phylogenetic trees of the SX10 strain. Phylogenetic trees based on the complete nucleotide sequences of 856 PiCV **(A)** and the amino acids of the Rep protein **(B)** from GenBank were aligned with MAFFT v7.525 and constructed via IQ-TREE v2.3.6.

Supplementary Figure S3 Identification of PiCV Cap in group A–E strains via the mAb 1G6-4C4. The *cap* genes from the group A strain (GenBank No. PQ472729.1), group B strain (GenBank No. OQ715331.1), group D strain (GenBank No. OR843262.1) and group E strain (GenBank No. MW181971.1) were synthesized into pcDNA3-2×Flag. The plasmid was transfected into HEK293T cells. IFA assay **(A)** and Western blotting **(B)** for the mAb 1G6-4C4. A mouse anti-DDDDK-Tag mAb was used as a positive control.

## References

[ref1] Cságola A. Lorincz M. Tombácz K. Wladár Z. Kovács E. Tuboly T. (2012). Genetic diversity of pigeon circovirus in Hungary. Virus Genes 44, 75–79. doi: 10.1007/s11262-011-0669-6, PMID: 21922293

[ref2] Duchatel J. P. Todd D. Smyth J. A. Bustin J. C. Vindevogel H. (2006). Observations on detection, excretion and transmission of pigeon circovirus in adult, young and embryonic pigeons. Avian Pathol. 35, 30–34. doi: 10.1080/03079450500465692, PMID: 16448939

[ref3] Duchatel J. P. Todd D. Willeman C. Losson B. (2009). Quantification of pigeon circovirus in serum, blood, semen and different tissues of naturally infected pigeons using a real-time polymerase chain reaction. Avian Pathol. 38, 143–148. doi: 10.1080/03079450902737805 19322713

[ref4] Duffy S. Holmes E. C. (2008). Phylogenetic evidence for rapid rates of molecular evolution in the single-stranded DNA begomovirus tomato yellow leaf curl virus. J. Virol. 82, 957–965. doi: 10.1128/JVI.01929-07, PMID: 17977971 PMC2224568

[ref5] Freick M. Müller H. Raue R. (2008). Rapid detection of pigeon herpesvirus, fowl adenovirus and pigeon circovirus in young racing pigeons by multiplex PCR. J. Virol. Methods 148, 226–231. doi: 10.1016/j.jviromet.2007.11.003, PMID: 18215428

[ref6] Gai W. Zheng W. Zhao Z. Wong G. Sun P. Yan L.. (2020). Assembly of pigeon circovirus-like particles using baculovirus expression system. Microb. Pathog. 139:103905. doi: 10.1016/j.micpath.2019.103905, PMID: 31790792

[ref7] Galfrè G. Milstein C. (1981). Preparation of monoclonal antibodies: strategies and procedures. Methods Enzymol. 73, 3–46. doi: 10.1016/0076-6879(81)73054-4 7300683

[ref8] Harzer M. Heenemann K. Sieg M. Vahlenkamp T. Freick M. Rückner A. (2021). Prevalence of pigeon rotavirus infections: animal exhibitions as a risk factor for pigeon flocks. Arch. Virol. 166, 65–72. doi: 10.1007/s00705-020-04834-w, PMID: 33067650 PMC7815556

[ref9] Huang Y. L. Castaneda O. A. Thongchan D. Khatri-Chhetri R. Tsai S. S. Wu H. Y. (2017). Pigeon circovirus infection in disqualified racing pigeons from Taiwan. Avian Pathol. 46, 359–366. doi: 10.1080/03079457.2017.1284305 28132523

[ref10] Katoh K. Standley D. M. (2013). MAFFT multiple sequence alignment software version 7: improvements in performance and usability. Mol. Biol. Evol. 30, 772–780. doi: 10.1093/molbev/mst010, PMID: 23329690 PMC3603318

[ref11] Khalifeh A. Kraberger S. Dziewulska D. Varsani A. Stenzel T. (2021). A pilot study investigating the dynamics of pigeon circovirus recombination in domesticated pigeons housed in a single loft. Viruses 13:964. doi: 10.3390/v13060964, PMID: 34067378 PMC8224587

[ref12] Lai G. H. Lin Y. C. Tsai Y. L. Lien Y. Y. Lin M. K. Chen H. J.. (2014). High yield production of pigeon circovirus capsid protein in the *E. coli* by evaluating the key parameters needed for protein expression. BMC Vet. Res. 10:115. doi: 10.1186/1746-6148-10-115, PMID: 24886262 PMC4046012

[ref13] Li X. Wang S. Li W. Wang S. Qin X. Wang J.. (2024). Investigating pigeon circovirus infection in a pigeon farm: molecular detection, phylogenetic analysis and complete genome analysis. BMC Genomics 25:369. doi: 10.1186/s12864-024-10303-4, PMID: 38622517 PMC11020411

[ref14] Loiko M. R. Junqueira D. M. Varela A. P. M. Tochetto C. Scheffer C. M. Lima D. A.. (2018). Columbid circoviruses detected in free ranging pigeons from southern Brazil: insights on PiCV evolution. Arch. Virol. 163, 3083–3090. doi: 10.1007/s00705-018-3990-8, PMID: 30105520

[ref15] Mankertz A. Hattermann K. Ehlers B. Soike D. (2000). Cloning and sequencing of columbid circovirus (coCV), a new circovirus from pigeons. Arch. Virol. 145, 2469–2479. doi: 10.1007/s007050070002, PMID: 11205099

[ref16] Minh B. Q. Schmidt H. A. Chernomor O. Schrempf D. Woodhams M. D. von Haeseler A.. (2020). IQ-TREE 2: new models and efficient methods for phylogenetic inference in the genomic era. Mol. Biol. Evol. 37, 1530–1534. doi: 10.1093/molbev/msaa015, PMID: 32011700 PMC7182206

[ref17] Muhire B. M. Varsani A. Martin D. P. (2014). SDT: a virus classification tool based on pairwise sequence alignment and identity calculation. PLoS One 9:e108277. doi: 10.1371/journal.pone.0108277, PMID: 25259891 PMC4178126

[ref18] Nath B. K. Das S. Das T. Forwood J. K. Raidal S. R. (2022). Development and applications of a TaqMan based quantitative real-time PCR for the rapid detection of pigeon circovirus (PiCV). J. Virol. Methods 308:114588. doi: 10.1016/j.jviromet.2022.114588, PMID: 35870671

[ref19] Raue R. Schmidt V. Freick M. Reinhardt B. Johne R. Kamphausen L.. (2005). A disease complex associated with pigeon circovirus infection, young pigeon disease syndrome. Avian Pathol. 34, 418–425. doi: 10.1080/03079450500267825, PMID: 16236576

[ref20] Sarker S. Patterson E. I. Peters A. Baker G. B. Forwood J. K. Ghorashi S. A.. (2014). Mutability dynamics of an emergent single stranded DNA virus in a naïve host. PLoS One 9:e85370. doi: 10.1371/journal.pone.0085370, PMID: 24416396 PMC3885698

[ref21] Standardization Administration of China. GB/T 16550–2020 Diagnostic techniques for Newcastle disease. (2020). China Standards Press.

[ref22] Stenzel T. Dziewulska D. Łukaszuk E. Custer J. M. De Koch M. D. Kraberger S.. (2024). The pigeon circovirus evolution, epidemiology and interaction with the host immune system under one loft race rearing conditions. Sci. Rep. 14:13815. doi: 10.1038/s41598-024-64587-3, PMID: 38877168 PMC11178769

[ref23] Stenzel T. Dziewulska D. Śmiałek M. Tykałowski B. Kowalczyk J. Koncicki A. (2019). Comparison of the immune response to vaccination with pigeon circovirus recombinant capsid protein (PiCV rCP) in pigeons uninfected and subclinically infected with PiCV. PLoS One 14:e0219175. doi: 10.1371/journal.pone.0219175, PMID: 31251772 PMC6599111

[ref24] Stenzel T. Dziewulska D. Tykałowski B. Śmiałek M. Kowalczyk J. Koncicki A. (2018). Immunogenicity of pigeon circovirus recombinant capsid protein in pigeons. Viruses 10:596. doi: 10.3390/v10110596, PMID: 30384424 PMC6265742

[ref25] Stenzel T. Pestka D. Choszcz D. (2014). The prevalence and genetic characterization of *Chlamydia psittaci* from domestic and feral pigeons in Poland and the correlation between infection rate and incidence of pigeon circovirus. Poult. Sci. 93, 3009–3016. doi: 10.3382/ps.2014-04219, PMID: 25306457

[ref26] Stenzel T. A. Pestka D. Tykałowski B. Śmiałek M. Koncicki A. (2012). Epidemiological investigation of selected pigeon viral infections in Poland. Vet. Rec. 171:562. doi: 10.1136/vr.100932, PMID: 23118041

[ref27] Stenzel T. Woźniakowski G. Pestka D. Choszcz D. Tykałowski B. Śmiałek M.. (2017). Application of pigeon circovirus recombinant capsid protein for detecting anti-PiCV antibodies in the sera of asymptomatic domestic pigeons and the potential use of a combination of serological and molecular tests for controlling circovirus infections in pigeon breeding flocks. Poult. Sci. 96, 303–308. doi: 10.3382/ps/pew266, PMID: 27578880

[ref28] Todd D. (2004). Avian circovirus diseases: lessons for the study of PMWS. Vet. Microbiol. 98, 169–174. doi: 10.1016/j.vetmic.2003.10.010, PMID: 14741130

[ref29] Todd D. Duchatel J. P. Bustin J. C. Scullion F. T. Scullion M. G. Scott A. N.. (2006). Detection of pigeon circovirus in cloacal swabs: implications for diagnosis, epidemiology and control. Vet. Rec. 159, 314–317. doi: 10.1136/vr.159.10.314, PMID: 16950888

[ref30] Todd D. Duchatel J. P. Weston J. H. Ball N. W. Borghmans B. J. Moffett D. A.. (2002). Evaluation of polymerase chain reaction and dot blot hybridisation tests in the diagnosis of pigeon circovirus infections. Vet. Microbiol. 89, 1–16. doi: 10.1016/s0378-1135(02)00154-2, PMID: 12223158

[ref31] Tsai S. S. Chang Y. L. Huang Y. L. Liu H. J. Ke G. M. Chiou C. J.. (2014). Development of a loop-mediated isothermal amplification method for rapid detection of pigeon circovirus. Arch. Virol. 159, 921–926. doi: 10.1007/s00705-013-1906-1, PMID: 24193953

[ref32] Yamamoto E. Ito H. Kitamoto E. Morinishi K. Yano A. Miyoshi S.. (2015). Complete genome sequence of pigeon circovirus detected in racing pigeons in western Japan. Virus Genes 51, 140–143. doi: 10.1007/s11262-015-1211-z, PMID: 26066052

[ref33] Yu X. Liu X. Zheng X. Wu D. Liu J. Zhu C. (2007). Cloning and prokaryotic expression of the △cap gene of pigeon circovirus isolated from Zhejiang province. Chin. J. Prev. Vet. Med. 29, 680–684. doi: 10.3969/j.issn.1008-0589.2007.09.006

[ref34] Yu X. Zhu C. Zheng X. Mu A. Yu H. (2009). Cloning and analysis of the complete genomes of pigeon circovirus from Zhejiang province. Chin. J. Virol. 25, 355–361. doi: 10.13242/j.cnki.bingduxuebao.002022 19954112

[ref35] Zhang Z. Dai W. Wang S. Dai D. (2015). Epidemiology and genetic characteristics of pigeon circovirus (PiCV) in eastern China. Arch. Virol. 160, 199–206. doi: 10.1007/s00705-014-2255-4, PMID: 25348272

[ref36] Zhang Z. Lu C. Wang Y. Wang S. Dai D. Chen Z.. (2011). Molecular characterization and epidemiological investigation of pigeon circovirus isolated in eastern China. J. Vet. Diagn. Invest. 23, 665–672. doi: 10.1177/1040638711407878 21908307

