# Supplementary figures and images for: A pan-genotypic indirect competitive ELISA for serological detection of pigeon circovirus antibodies
Source: Front Microbiol. 2025 Jul 30;16:1612715. doi: 10.3389/fmicb.2025.1612715 (PMC12343533; doi:10.3389/fmicb.2025.1612715)

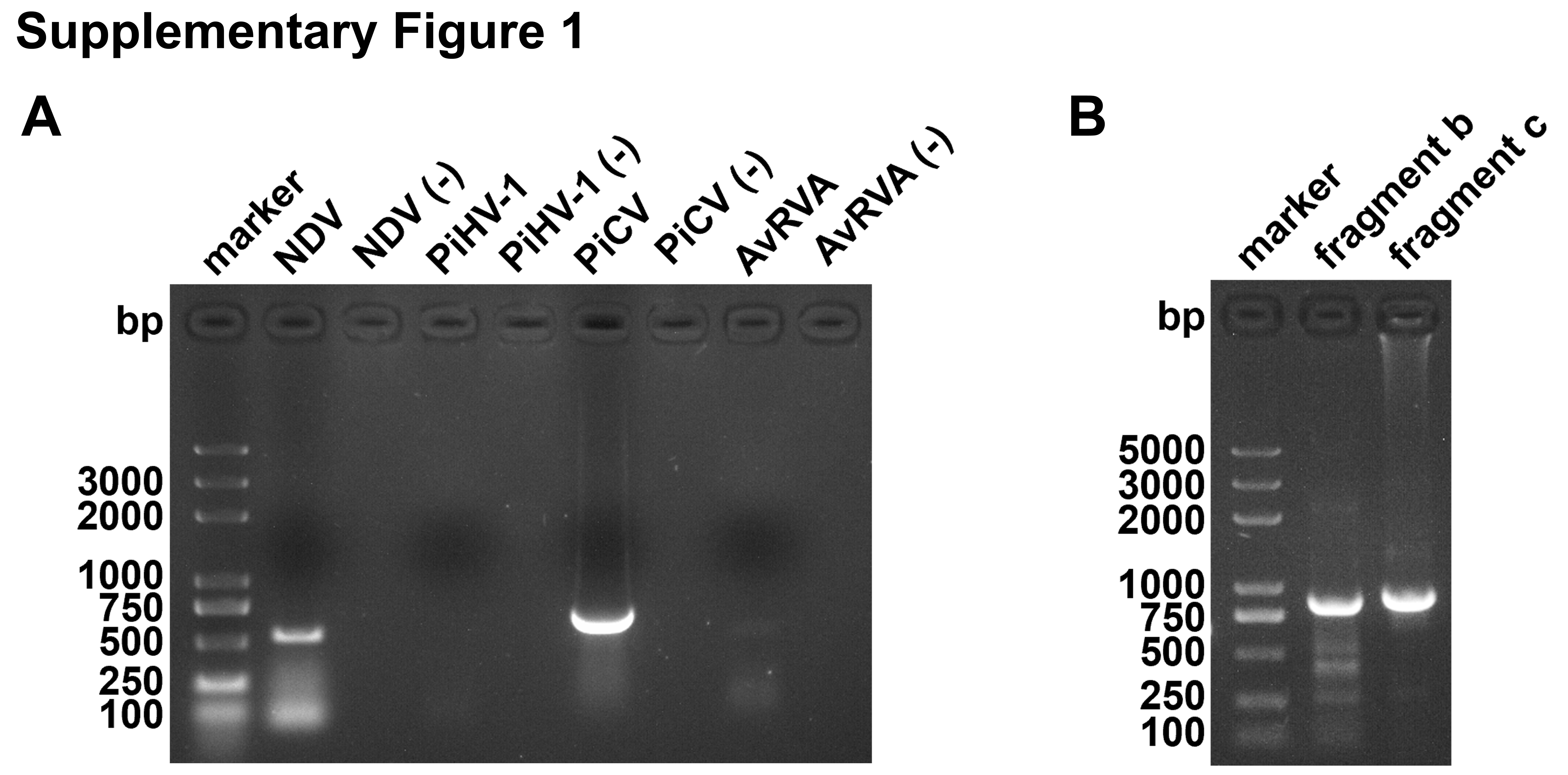

Supplement: Supplementary Figure S1 — PCR amplification of target genes. (A) PCR amplification of viral genes. NDV, Newcastle disease virus; PiHV-1, pigeon herpesvirus 1; PiCV, pigeon circovirus; AvRVA, avian rotavirus A. “-” indicates that the primers were designed in this research. (B) PCR amplification of PiCV gene fragments. [file Image_1.tif]

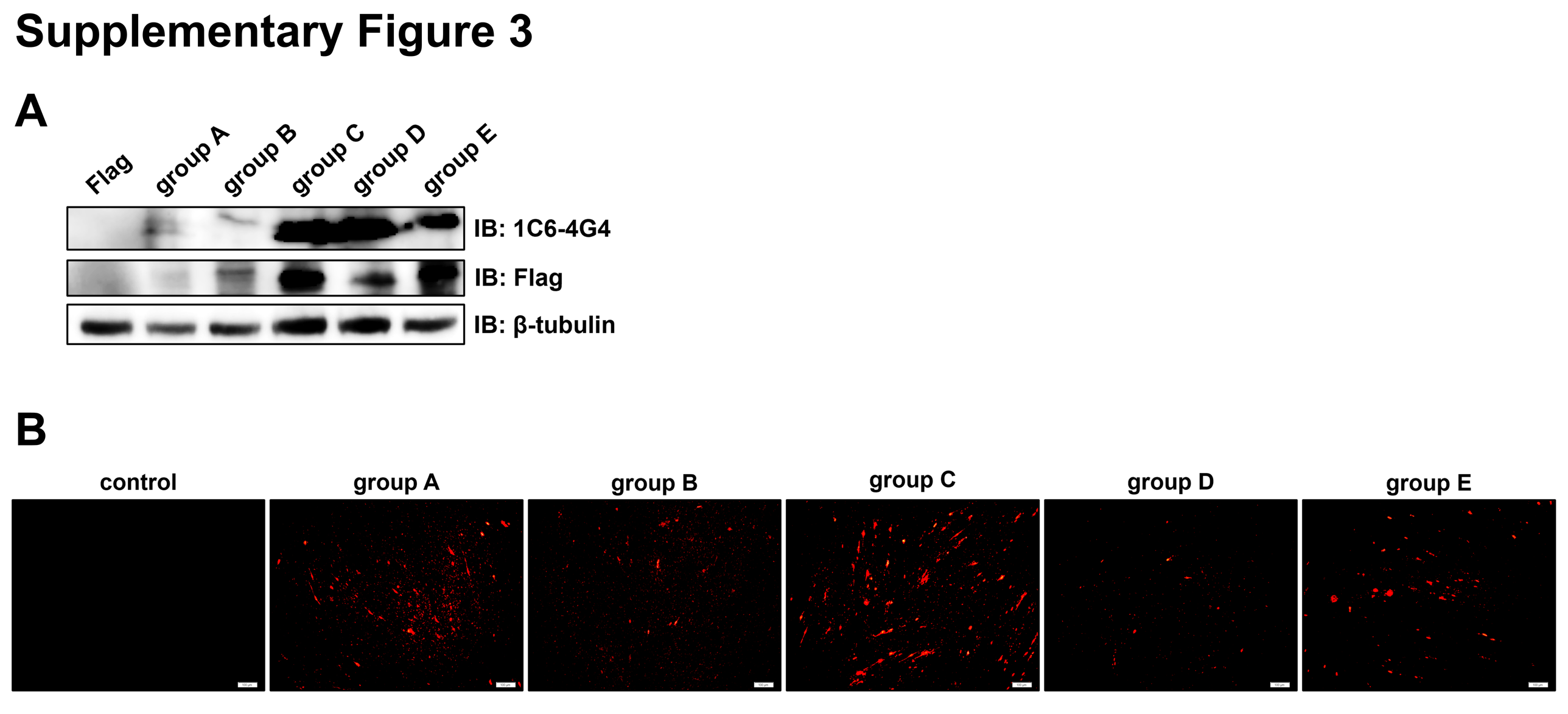

Supplement: Supplementary Figure S3 — Identification of PiCV Cap in group A–E strains via the mAb 1G6-4C4. The cap genes from the group A strain (GenBank No. PQ472729.1), group B strain (GenBank No. OQ715331.1), group D strain (GenBank No. OR843262.1) and group E strain (GenBank No. MW181971.1) were synthesized into pcDNA3-2×Flag. The plasmid was transfected into HEK293T cells. IFA assay (A) and Western blotting (B) for the mAb 1G6-4C4. A mouse anti-DDDDK-Tag mAb was used as a positive control. [file Image_3.tif]
