# Supplementary material for: A pan-genotypic indirect competitive ELISA for serological detection of pigeon circovirus antibodies
Source: Front Microbiol. 2025 Jul 30;16:1612715. doi: 10.3389/fmicb.2025.1612715 (PMC12343533; doi:10.3389/fmicb.2025.1612715)
Supplement: Supplementary file 5 [file Table_2.docx]

Supplementary Table 2. Intra-batch repeatability detection of the icELISA.

| Serum number | Intra-batch | | | Mean | SD | CV |
| --- | --- | --- | --- | --- | --- | --- |
| 1 | 0.577 | 0.629 | 0.576 | 0.594 | 0.025 | 4.17% |
| 2 | 0.399 | 0.401 | 0.378 | 0.393 | 0.010 | 2.65% |
| 3 | 0.448 | 0.476 | 0.459 | 0.461 | 0.012 | 2.50% |
| 4 | 1.056 | 1.102 | 1.077 | 1.078 | 0.019 | 1.74% |
| 5 | 0.987 | 0.949 | 1.031 | 0.989 | 0.034 | 3.39% |
| 6 | 0.871 | 0.796 | 0.847 | 0.838 | 0.031 | 3.73% |

SD, standard deviations; CV, the coefficient of variation.
