# Supplementary material for: A pan-genotypic indirect competitive ELISA for serological detection of pigeon circovirus antibodies
Source: Front Microbiol. 2025 Jul 30;16:1612715. doi: 10.3389/fmicb.2025.1612715 (PMC12343533; doi:10.3389/fmicb.2025.1612715)
Supplement: Supplementary file 6 [file Table_3.docx]

Supplementary Table 3. Inter-batch repeatability detection of the icELISA.

| Serum number | Inter-batch | | | Mean | SD | CV |
| --- | --- | --- | --- | --- | --- | --- |
| 1 | 0.498 | 0.557 | 0.601 | 0.552 | 0.042 | 7.64% |
| 2 | 0.387 | 0.446 | 0.398 | 0.410 | 0.026 | 6.24% |
| 3 | 0.476 | 0.443 | 0.411 | 0.443 | 0.027 | 5.99% |
| 4 | 1.091 | 1.247 | 1.221 | 1.186 | 0.068 | 5.75% |
| 5 | 1.045 | 0.918 | 0.974 | 0.979 | 0.052 | 5.31% |
| 6 | 0.849 | 0.877 | 1.014 | 0.913 | 0.072 | 7.89% |

SD, standard deviations; CV, the coefficient of variation.
