# Supplementary material for: A pan-genotypic indirect competitive ELISA for serological detection of pigeon circovirus antibodies
Source: Front Microbiol. 2025 Jul 30;16:1612715. doi: 10.3389/fmicb.2025.1612715 (PMC12343533; doi:10.3389/fmicb.2025.1612715)
Supplement: Supplementary file 7 [file Table_4.docx]

Supplementary Table 4. Intra-batch repeatability detection of iELISA.

| Serum number | Intra-batch | | | Mean | SD | CV |
| --- | --- | --- | --- | --- | --- | --- |
| 1 | 1.432 | 1.466 | 1.512 | 1.470 | 0.033 | 2.23% |
| 2 | 1.923 | 1.778 | 1.889 | 1.863 | 0.062 | 3.32% |
| 3 | 1.547 | 1.631 | 1.592 | 1.590 | 0.034 | 2.16% |
| 4 | 0.167 | 0.156 | 0.162 | 0.162 | 0.004 | 2.78% |
| 5 | 0.287 | 0.267 | 0.273 | 0.276 | 0.008 | 3.08% |
| 6 | 0.194 | 0.207 | 0.196 | 0.199 | 0.006 | 2.87% |

SD, standard deviations; CV, the coefficient of variation.
