# Supplementary material for: A pan-genotypic indirect competitive ELISA for serological detection of pigeon circovirus antibodies
Source: Front Microbiol. 2025 Jul 30;16:1612715. doi: 10.3389/fmicb.2025.1612715 (PMC12343533; doi:10.3389/fmicb.2025.1612715)
Supplement: Supplementary file 8 [file Table_5.docx]

Supplementary Table 5. Inter-batch repeatability detection of iELISA.

| Serum number | Inter-batch | | | Mean | SD | CV |
| --- | --- | --- | --- | --- | --- | --- |
| 1 | 1.547 | 1.429 | 1.490 | 1.489 | 0.048 | 3.24% |
| 2 | 1.979 | 1.708 | 1.884 | 1.857 | 0.112 | 6.05% |
| 3 | 1.447 | 1.639 | 1.599 | 1.562 | 0.083 | 5.30% |
| 4 | 0.181 | 0.159 | 0.183 | 0.174 | 0.011 | 6.24% |
| 5 | 0.293 | 0.262 | 0.271 | 0.275 | 0.013 | 4.73% |
| 6 | 0.169 | 0.199 | 0.178 | 0.182 | 0.013 | 6.91% |

SD, standard deviations; CV, the coefficient of variation.
