# Supplementary material for: A pan-genotypic indirect competitive ELISA for serological detection of pigeon circovirus antibodies
Source: Front Microbiol. 2025 Jul 30;16:1612715. doi: 10.3389/fmicb.2025.1612715 (PMC12343533; doi:10.3389/fmicb.2025.1612715)
Supplement: Supplementary file 9 [file Table_6.docx]

Supplementary Table 6. The icELISA clinical sample test results.

| Serum number | PI (%) | Result |
| --- | --- | --- |
| 1 | 67.27 | P |
| 2 | 53.49 | P |
| 3 | 59.90 | P |
| 4 | 6.41 | N |
| 5 | 54.55 | P |
| 6 | 53.01 | P |
| 7 | 77.99 | P |
| 8 | 9.09 | N |
| 9 | 53.49 | P |
| 10 | 68.71 | P |
| 11 | 14.64 | N |
| 12 | 68.04 | P |
| 13 | 44.31 | P |
| 14 | 13.11 | N |
| 15 | 20.86 | N |
| 16 | 3.54 | N |
| 17 | 23.16 | N |
| 18 | 32.06 | P |
| 19 | 52.06 | P |
| 20 | 9.47 | N |
| 21 | 6.32 | N |
| 22 | 49.09 | P |
| 23 | 26.32 | N |
| 24 | 42.97 | P |
| 25 | 16.65 | N |
| 26 | 30.53 | P |
| 27 | 6.70 | N |
| 28 | 20.00 | N |
| 29 | 18.09 | N |

PI, percentage of inhibition; P, positive; N, negative.
