# Supplementary material for: A pan-genotypic indirect competitive ELISA for serological detection of pigeon circovirus antibodies
Source: Front Microbiol. 2025 Jul 30;16:1612715. doi: 10.3389/fmicb.2025.1612715 (PMC12343533; doi:10.3389/fmicb.2025.1612715)
Supplement: Supplementary file 10 [file Table_7.docx]

Supplementary Table 7. The iELISA clinical sample test results.

| Serum number | OD_450_ | Result | Serum number | OD_450_ | Result | Serum number | OD_450_ | Result |
| --- | --- | --- | --- | --- | --- | --- | --- | --- |
| 1 | 0.862 | P | 11 | 0.343 | N | 21 | 0.249 | N |
| 2 | 0.572 | P | 12 | 0.797 | P | 22 | 0.517 | P |
| 3 | 0.624 | P | 13 | 0.365 | N | 23 | 0.329 | N |
| 4 | 0.297 | N | 14 | 0.263 | N | 24 | 0.427 | P |
| 5 | 0.539 | P | 15 | 0.268 | N | 25 | 0.359 | N |
| 6 | 0.664 | P | 16 | 0.296 | N | 26 | 0.426 | P |
| 7 | 1.547 | P | 17 | 0.264 | N | 27 | 0.199 | N |
| 8 | 0.344 | N | 18 | 0.269 | N | 28 | 0.261 | N |
| 9 | 0.669 | P | 19 | 0.819 | P | 29 | 0.256 | N |
| 10 | 0.801 | P | 20 | 0.185 | N |  |  |  |

P, positive; N, negative.
